# The Evidence Relative to Dr. Jenner's Petition

**Published:** 1802-08-01

**Authors:** 


### *The Evidence relative to Dr. Jenner's Petition.*

Concluded from page 26 of the laft Number.

WE are happy to commence our promifed account of the evidence which feemed, or was intended, to oppofe Dr. Jenner's claim, by informing our Readers that he will receive the premium of TEN THOUSAND POUNDS clear of fees or any other deduction.

We are ftill more happy in being able to inform them, that the idea of a *general* fubfcription or contribution, (but more extenfive than that recommended by Dr. Beddoes, page 7,) had occurred to many perfons in the metropolis; we believe that a Committee is now forming to digeft a plan for conducting it, and our readers may depend on receiving the earlieft account of the progrefs.

We now proceed to the performance of our promife, and repeat again, that we are not acquainted with any adverfe evidence which the Committee have not publifhed in their Appendix, although they have omitted many of Dr. Jenner's explanations, and much of his rebutting evidence.

---

As Dr. MOSELEY, Phyfician to Chelfea Hofpital, (No. 30.) was known to have written againft the New Inoculation when when it firft became general, Dr. Jenner fuggefted to the Committee the propriety of examining him, which they readily agreed to do. Dr. M. gave his evidence with great candour, and totally free from that acrimony which appeared in fome other inftances. His principal reafon for oppofing the new Inoculation at that time, for he has long ceafed his oppofition, was his obferving it to be getting into improper hands. He faw but a few perfons properly qualified, and an immenfe number totally ignorant of medicine, rafhly, as he thought, communicating difeafes from brutes to the human fpecies, and wifhed to ftem this torrent of experimental mania, till perfons properly qualified had made the neceffary trials. Finding his efforts vain, he burnt his papers, and has not fince attended to the fubject. He could not refer the Committee to any adverfe cafe, nor to any perfons who had mentioned adverfe cafes to him, though he believed fuch cafes had been mentioned. He faid, if this Inoculation poffeffes all the advantages Dr. Jenner attributes to it, he has conferred a moft effential benefit upon mankind.

Mr. Birch, of St. Thomas's Hofpital, (No. 31,) mentioned the cafes fpoken to by Mr. Cline, (fee p. 17,) as proofs of the *inefficacy* of the new Inoculation; but faid it was a fubject he had not much attended to, as he did not like it, and as it was not the cuftom of the Hofpital for the Medical Officers to interfere with each other's patients.

Dr. Rowley, Phyfician to the Mary-le-bone Infirmary, related the Oxford cafes, which made much noife at the time, as proofs of the *inefficacy* of the Vaccine Inoculation; but this matter was completely cleared up by the evidence of Dr. Wall, of Oxford, and others; by which it appeared, that thefe children had never gone through the vaccine difeafe. Indeed, when we confider how many people, who were ignorant of the appearance of the true vaccine puftule, ventured to inoculate at that time, and how many never faw their patients after they had inferted the virus, we muft be aftonifhed that fo few accidents of that kind have occurred. It appeared to us that both Dr. Rowley and Mr. Birch oppofed the New Inoculation, not from any knowledge they had of the fubject, (they confeffed they had never thought it worth their attention) but from a fondnefs each entertained for certain modes of conducting the Variolous Inoculation, which he believed to be peculiar to himfelf. For they more than infinuated, that if any perfon died, or was in danger from inoculated Small-Pox, it muft always be through the faults of the Inoculator or patient, as they were never in danger of lofing any; and therefore there was *no need* of the new difcovery.

The

The only adverſe caſe that appeared to us to merit any attention, was the caſe of George Clarke, a marine, of Portſmouth. It ſeems pretty evident, that he had the ſmall-pox while the Committee were ſitting; though many perſons at that time believed it to be the chicken-pox. But there was no proof of his ever having gone through the cow-pox; on the contrary, it is certain he never had that diſeaſe, though it is confeſſed he had been inoculated with that intention about three years before, by a reſpectable practitioner of Portſmouth, who commenced that practice with him, and two or three others. Now, if in this caſe we admit that he had the true vacciola, which is not true, and the ſmall-pox three years afterwards, what is one caſe againſt *two millions?*

When the Inoculation of the ſmall-pox began to be practiſed generally in this kingdom, many inſtances occurred of perſons taking the ſmall-pox afterwards; and yet no one now doubts the preventive power of the inoculated variola.

---

Such is the amount of the evidence *againſt the certainty of the protection* which vaccination affords from the ſmall-pox infection.

There was no evidence againſt the importance of ſuperſeding the ſmall-pox; againſt the mildneſs of the cow-pox; againſt its never prediſpoſing to or exciting other diſeaſes in the conſtitution, which the mildeſt form of ſmall-pox is often ſeen to do; againſt its being communicable only by inoculation; or, in ſhort, againſt any of the points alleged by Dr. Jenner, unleſs the following may be deemed ſuch.

But before we give Dr. Pearſon's evidence, we wiſh to obſerve, that no important diſcovery has ever been made in which the Diſcoverer has not been denied the merit of originality. Nothing is ſo eaſy as to attack an author on this ground. When Harvey diſcovered the circulation, the Galeniſts aſſerted that it was nothing new, and that many proofs of his knowledge of it, were to be found even in the works of Galen. There is ſomething very ſimilar in the conduct of Dr. Harvey and Dr. Jenner; each of them compleated his Diſcovery, and then publiſhed ſix or ſeven experiments to demonſtrate the truth of their concluſions. In giving the evidence brought forward by Dr. Pearſon, with a view to overturn Dr. Jenner's claim to originality, we ſhall be as careful as poſſible to ſtate nothing but what we believe to be correct; but if we ſhould fall into any ſlight inaccuracy, we ſhall always be ready to correct it whenever pointed out.

When Dr. P. was feated, and afked the ufual introductory queftion, viz.

"Are you acquainted with Vaccine Inoculation, and what is your opinion of it?"

He delivered an oration in which he depreciated Dr. Jenner's merit as much as poffible, and extolled himfelf. As all this was foreign to the purpofe, it was not taken down, and the Committee adjourned. On the morrow he came to hear his evidence read, and to correct any inaccuracies in it; but to his great aftonifhment, the whole oration was omitted, and he was informed that nothing could be taken down by the clerk unlefs dictated flowly, that he might have time to write fairly and legibly.

He then gave the following evidence; and as we wifh to avoid the cenfure he has paffed on the Committee, we fhall give the whole, verbatim et literatim, as approved by himfelf.

---

Mercurii, 14 Aprilis, 1802.

Admiral BERKELEY in the Chair.

Dr. PEARSON called in and examined, and ftated, That Dr. Heberden authorized him to ftate, on the authority of Dr. Lynd and Mr. Battifcombe of Windfor, that there is now living near Windfor a perfon who many years ago (the fon of an apothecary) was inoculated by his father for the cow-pox. *

Did Dr. Heberden inform you whether this inoculation was performed from one human being to another, or from the virus taken immediately from the cow?

That is a queftion I cannot anfwer.

What farther facts do you know affecting Dr. Jenner's claim of being the promulgator or inventor of vaccine inoculation?

I have admitted Dr. Jenner was the gentleman who firft fet on foot the inquiry into the advantages of vaccine inoculation, but I apprehend that the practice of vaccine inoculation, which was firft promulgated by Dr. Jenner, has been eftablifhed almoft entirely by other practitioners, and that the new facts, or which I confider to be new, have been in my opinion difproved by fubfequent obfervers; and that in confequence of thofe facts being difproved, together with the very extenfive experience of other perfons, we owe the prefent extenfive practice of the vaccine inoculation.

Will you inform the Committee, who thofe practitioners and perfons are to whom you refer? The

---

* How far this is true will be feen in the evidence of thefe gentlemen.

The cow-pox inoculation after Dr. Jenner's book was published in May or June, 1798, was not practiced after Dr. Jenner's feventh or eighth cafe contained in his work the whole refult of his experience, by any perfon that I know of, till January, 1799, neither Dr. Jenner nor any perfon that I could find being in poffeffion of matter; but in January, 1799, in confequence of a general inquiry, which I had inftituted immediately after Dr. Jenner's publication, information was given of the cow-pox difeafe breaking out in two of the cow-ftables near London, and from thefe fources Dr. Woodville and myfelf collected matter, by which, in the courfe of about three months, not fewer, I think, than 300 perfons were inoculated for the cow-pox in addition to the feven or eight cafes of Dr. Jenner, then the whole ftock of facts of inoculation before the public. Befides carrying on the inoculation ourfelves in this manner, we diffeminated the matter throughout the country, in particular to Dr. Jenner himfelf, and particularly alfo, I within that time iffued a printed letter, directed to upwards of two hundred practitioners in different parts of the kingdom, containing thread impregnated with cow-pox matter. In the courfe of this practice we already learnt that *young infants* might be inoculated with fafety, which I confidered to be then a new fact, Dr. Jenner not having had the experience, and being apprehenfive of ferious confequences from inoculating them. Secondly, That the inoculated arms, fo far from requiring cauftic or efcarotic, or other topical applications, were fooner cured than in the inoculated fmall-pox; that Dr. Woodville's publication in June, 1799, appeared, containing the cafes of upwards of four hundred inoculated in that time; and in Auguft, 1799, I publifhed a ftatement of inoculation, referring to many practitioners, who had furnifhed me with reports of inoculation, with matter which I myfelf had furnifhed; among thefe I beg leave to mention Mr. Kelfon of Seven Oaks, Dr. Mitchel of Chatham, and Dr. Harrifon's cafes, as communicated to me by Sir Jofeph Banks, and by that time I had alfo introduced it into the army, through the hands of the Surgeon General Mr. Keate, and reports frequently came into my hands by his direction from the army; I had alfo by that time introduced the vaccine inoculation into many parts of the continent, and received reports of the fuccefsful practice of it, in particular from Dr. De Carro of Vienna; in addition to thefe teftimonies contained in the paper above alluded to, is the refult of my own practice in three parifhes of poor people inoculated under my fuperintendance, fo that, in that paper, I believe it will be found that two thoufand cafes had by that time been afforded for the public by Dr. Woodville and myfelf, and the perfons with whom I was in correfpondence, and who are mentioned in the papers alluded to; by this time too, fome difficulties appear to have been removed in a great meafure by fome facts ftated to the public by Dr. Jenner, in particular that I publifhed experiments of inoculation in the paper alluded to of inoculating perfons with the cow-pox, who had undergone the fmall-pox, to fhew that they *could not take the cow-pox after the small-pox*, contrary to Dr. Jenner. Secondly, that experiments to fhew that perfons could not take the fmall-pox both locally and conftitutionally, who had already gone through the cow-pox, alfo contrary to Dr. Jenner. Thirdly, many perfons had by this time, made experiments to fhew that the *cow-pox did not originate in the grease of horses' heels*, as Dr. Jenner had afferted; thefe fentiments will be found in a printed ftatement which I beg to deliver in, as publifhed by me. In the fpring of the year 1799, while the above ftated evidence was collected, a fecond publication appeared from Dr. Jenner, adding nothing but a few cafes of inoculation further of the cow-pox, but recommending cauftic or efcarotic applications to the inoculated parts, in the cow-pox, not found neceffary by the medical perfons alluded to in my evidence; and I confider that the diftinctive characters of the cow-pox were underftood better by fome of the above alluded to perfons than by Dr. Jenner. The vaccine inoculation was next confiderably eftablifhed by the Cow-pox Inftitution, of which I was one of the founders, commencing at the very clofe of the year 1799; which inftitution has been the principal office, I apprehend, for fupplying the world in general, and the army and navy in particular, with matter; and where a regular regifter is kept of each of the cafes inoculated, more fully and accurately than had been done any where before or fince that time; where the authenticity of the cafes, from the nature of the inftitution, is eftablifhed in a manner that I apprehend will be confidered as unexceptionable; this appears from a regifter of about feven hundred cafes already entered, and open to the infpection of the fubfcribers. By this time, namely, the clofe of the year 1799, I think I can make it appear that about four thoufand perfons had been inoculated by Dr. Woodville, myfelf, and correfpondents, which can be referred to. I here clofe my evidence, as I confider it of very fmall importance, comparatively, what was done by others after this time, all the facts that I recollect of ufe in practice being by this time eftablifhed, as they have been fince confirmed.*

Did

* Our readers will collect the fubftance of the oration from the above evidence. We make no commentary on the remaining anfwers.

Did you never hear of inoculation having been performed by Mr. Cline, with matter furnifhed by Dr. Jenner, previous to the time you began to practice vaccine inoculation?

I cannot recollect diftinctly.

Were not the feven or eight cafes of Dr. Jenner, alluded to by you, cafes of inoculation from one human being to another?

Some of them were, others were not.

Had not many, or a large majority of your firft cafes, variolous-like eruptions?

The matter which had never been in the Small-pox Hofpital, and which I myfelf took from the cows at the two cow-ftables above alluded to, fcarcely ever afforded any eruptions like the fmall-pox; but when I obtained matter to fupply my correfpondents in the country, not having enough of my own, but obtained it from the Small-pox Hofpital, it frequently, according to the reports of my correfpondents, and in a few cafes where I ufed it myfelf, did produce fuch eruptions.

Was not the matter or virus which you diftributed, found great fault with, on account of the eruptions it produced?

No, it was not found fault with; but many people were difappointed, as they expected that one of the advantages attending the inoculation was to be exempt from eruptions.

Did not thefe eruptions, which were produced by your matter, very much difcourage practitioners and the public, and very much retard the progrefs of the new inoculation?

I fhould think it did not.

Do you not know there is a cafe in Dr. Jenner's firft publication of his having inoculated a child of eleven months old?

I believe there is one cafe.

Did not Dr. Woodville and yourfelf take the vaccine matter in Gray's Inn Lane, for the purpofe of commencing your experiments, from a perfon fully marked with the fmall pox?

No fuch cafe is in my recollection.

Have thofe facts ftated by you to militate againft Dr. Jenner's declared opinions remained uncontradicted by him? Does he ftill maintain them, or has he publicly retracted them?

I think he has not retracted them.—Withdrew.

Adjourned till to-morrow.

When any thing important happened to fpring up in the evidence, which the witnefs himfelf could not fpeak to, the Committee, in general, made it a rule to fummon the proper perfons as foon as poffible.

As Dr. Heberden's authority had been mentioned in proof of the practice of vaccine inoculation many years ago, he was fummoned for the morrow.

When the Committee met, on the 15th, Dr. Pearſon, having a bundle of papers in his hand, begged to be heard, ſtating, that theſe papers contained deciſive proof of the practice of Vaccine Inoculation, long before Dr. Jenner's diſcovery; and that he received them from Mr. Keate. This opened a new field for inquiry into the authenticity of the papers, the author of them, their contents, whether any part of them had ever been publiſhed, and whether Dr. J. had borrowed his ideas from the author. Dr. P. aſſerted, that they contained every thing to be found in Dr. Jenner's works, except his errors; and "I am," ſaid he, "convinced that the author was a very experienced inoculator, for I find one obſervation which I did not think had occurred to any body but myſelf." This attack on Dr. Jenner's originality of diſcovery, made a ſenſible impreſſion on all preſent; for it was concluded that no perſon of Dr. P's reſpectability would make ſuch aſſertions without plauſible proof, at leaſt, to ſupport them. Dr. Heberden entering the room about this time, his examination was commenced, as follows, viz.

Jovis, 15° die Aprilis, 1802.

Admiral BERKELEY in the Chair.

Dr. HEBERDEN called in and examined. The ſtatement made yeſterday, by Dr. Pearſon, being read,

Dr. Heberden ſtated, That all he knew upon the ſubject was, about three years ago Dr. Lynd of Windſor, mentioned to him in converſation, that there was living, near Windſor, a young man, apprentice to an apothecary, who, when a child, was inoculated with vaccine matter by his father, who was an apothecary in the Weſt of England; with reſpect to Mr. Batiſcombe, he could not ſpeak, having heard nothing of it.

Do you know his name?

No, I do not.

Did you underſtand it to have been an inoculation from the cow?

I am not certain whether it was from the cow, or ſome perſon who had caught the diſeaſe.—Withdrew.

Dr. Jenner finding it neceſſary to eſtabliſh the date of his diſcovery by evidence, requeſted Mr. Gardiner to be examined; (ſee page 19 of our laſt number) who proved that Dr. Jenner had publiſhed his diſcovery, among the medical men in his neighbourhood, as early as the ſummer of 1780. And he not only publiſhed the diſcovery, but he explained to his neighbours, neighbours his views and expectations almost as compleatly as he could do at present.

Dr. Jenner was proceeding to give evidence that the matter sent by Dr. P. and some others, from London into the country, had produced the small-pox instead of the cow-pox; which very much injured and retarded the new inoculation in many places. But the entrance of Mr. Keate determined the Committee to proceed with his examination.

Mr. KEATE, Surgeon-General to the Army, called in and examined.

Dr. Pearson having stated that you were in possession of some manuscripts which might tend to throw some light upon the discovery of vaccine inoculation, you are desired to produce them.

The papers left by Mr. Robert Keate, my nephew, who received them from the author's son relative to a variety of matter on the subject of inoculation: I have made extracts of those parts which relate to cow-pox, which I will identify from the original, and lay before the Committee.

Were the observations contained in those papers ever made public?

I do not know that they were.

Have you been much conversant in vaccine inoculation, and what is your opinion of it?

In the year 1799, by means of Dr. Pearson, seeing a number of his patients; afterwards inoculating with him the poor of several parishes; then introducing it to the army in all quarters of the globe; and lastly, giving my utmost assistance towards founding an institution for the benefit of the poor in London.

Have you not met with instances of a spurious sort of vaccine pustule?

I do not recollect that I have.

Have you met with such varieties in the disease as to make it difficult to determine whether the patient has had the perfect disease?

No, I do not know that I have: there are but two kinds in my judgment, one local and the other constitutional.

Do you think that a constitutional affection is essentially necessary to the perfect disorder, or that it consists in the regular progress of the pustule only?

I think it is the true characteristic of the disease; my opinion is, that the constitution should be affected more or less.

Would you venture to pronounce a patient safe from small-pox infection, in whom you had not been able to observe any constitutional affection whatever, though the progress of the local disease had been perfectly regular?

 Yes,

Yes; I think that the local appearances may be ſo ſtrong and evident as to warrant the concluſion on my part, that the conſtitutional ſymptoms might not be ſtrong enough to be obſerved.

Has your mode of practice varied ſince you firſt undertook this mode of practice?

No.

Do you know of any other humours or diſorders ſuppoſed to be excited by this mode of inoculation?

No.

Whom do you look upon as the Diſcoverer of this mode of inoculation.

I conſider Dr. Jenner to be the perſon to whom much merit is due in publiſhing the caſes of inoculation, which have given riſe to further inveſtigation and improvement; but to whom to attribute the diſcovery, I am unable to ſay.

In thoſe caſes is there not mention made of a child or children being inoculated?

I think that the youngeſt, inoculated by Dr. Jenner, as far as I can recollect, was eleven months old.

Did you ever hear of inoculation for the cow-pox before Dr. Jenner's publication?

I never did.

Have you heard ſince his publication, that any other perſon had promulgated, or practiſed, that mode of inoculation antecedent to that time?

I have heard that others had inoculated for the cow-pox from the animal, previous to that time.

Did you ever hear of this diſorder having been inoculated from one human being to another before Dr. Jenner's publication.

No.—Withdrew.

As Mr. Keate had received the papers from his nephew, it became neceſſary to ſummon him; and he being out of town, the Committee adjourned to the 26th.

---

Lunæ, 26° die Aprilis, 1802.

Mr. BANKS in the Chair.

Mr. ROBERT KEATE called in and examined.

Papers mentioned by Dr. Pearſon and Mr. Keate laid upon the table.

State what you know of theſe papers.

Mr. Naſh, the ſon of the author, put them into my hands, in the ſummer of 1800.

Do

Do you know at what time thefe papers were written? I have underftood, after the year 1781.—Withdrew.

Mr. THOMAS NASH called in and examined.

When did you deliver thefe papers to Mr. Keate?

In 1799, or 1800.

Do you know at what time they were written?

Not for certain, but between the years 1781 and 1785: By the date 1781, in which year I was inoculated; and the year 1785, in which my father died.

Do you know the hand writing to be your father's?

This is the writing which was put into my hands as his; I have never feen my father write.

When did you firft fee thefe papers?

In 1795 or 1796.

By whom were they put into your hands?

By Mr. Battifcombe.

What account did he give of thefe papers?

On the death of my father, they were fent by my mother to her brother, Mr. Battifcombe, a medical man; he returned them to me in 1795 or 1796; I fancy, without ever looking at them; they were fent with other manufcripts in the fame hand writing, to be made what ufe of I pleafed.

Did you underftand from him, that he had communicated the contents to any perfon whatever?

I have every reafon to believe he did not.

Did you ever underftand that you yourfelf were inoculated by your father with vaccine matter?

Not for certain; I have heard my mother fay, that at the time of my inoculation, my father was greatly taken up in the ftudy of the cow-pox, and made many experiments, but of what nature fhe did not know.

Did you ever hear her fpeak of any perfons whom fhe knew to have been inoculated by your father with vaccine matter?

Certainly not, his experiments were entirely kept fecret* from her.

How long have you kept thofe papers in your poffeffion?

They were delivered to me in 1795 or 1796, and then I delivered them to Mr. Keate in 1799 or 1800.

Did

---

* Suppofing the author of the papers to have known as much as Dr. P. ftated, which was far from being the cafe, yet, as he kept his knowledge a profound fecret, and as the papers were written three or four years after Dr. Jenner had publifhed his difcovery, they could not affect his claim if they had been publifhed.

Did you ever make any part of them public, or communicate the contents to any perfon in the mean while?

Never, till they were given to Mr Keate.

Have you any reafon to think that Dr. Jenner was acquainted with the author of thefe papers?

I never heard that he was till this morning, and then from rumour.

Who gave you this intelligence?

I heard it from Mr. R. Keate.—Withdrew.

Mr. ROBERT KEATE again called in and examined.

Have you any reafon to think that Dr. Jenner was acquainted with the author of thefe papers?

I heard from Mr. Battifcombe yefterday, that he believed he had heard Mr. Nafh and his fifter mention the name of Dr. Jenner; but was not at all certain that it was Dr. Jenner who applies to parliament.—Withdrew.

Adjourned till to-morrow.

---

Many of our Readers, no doubt, will be very defirous to know the contents of this Manufcript, on which fo much ftrefs has been laid; but as we were not favoured with the perufal of it, we can gratify them only by inferting thofe parts which the Committee have thought relevant to the inquiry before them.

We underftand that the chief object of the writer, was, to recommend fome improvements which he thought he had made in the inoculation of the *small-pox*. In his practice, as many others had done before him, he found that thofe who had taken the cow-pox by milking, or otherwife, would not take the inoculated fmall-pox.

Extracts from Manufcripts of Mr. Nafh, delivered to your Committee by Mr. Keate, as far as they relate to Cow Pox.

It is rather remarkable that no writer fhould have taken notice of the Cow Pox.

I never heard of one having the fmall-pox, who ever had the cow-pox. The cow-pox certainly prevents a perfon from having the fmall-pox.

I have now inoculated above fixty perfons who have been reported to have had the cow-pox, and I believe at leaft forty of them I could not infect with the variolous virus; the other twenty, or nearly that number, I think it very reafonable to prefume (as they were no judges) had not the real cow-pox. It is not my own opinion only, but that of feveral other medical cal gentlemen, that convinces me the cow-pox is a prophylactick for the ſmall-pox.

I have not been able to diſcover that the human ſpecies get it from the cows in any other manner than by contact with the parts immediately infected, ſuch as in milking; neither do I apprehend that one of the human ſpecies can communicate it to another, but by the ſame means, as I have known ſome of the inhabitants of a houſe where it was, eſcape it, but none of thoſe who lay in the ſame bed with a diſeaſed perſon.

In Mrs. Scammell and Mrs. Bracher, inoculation produced no eruption, no ſickneſs, and little or no ſuppuration of the arm, the place punctured not being bigger, when inflamed and ſuppurated, than a large pin's head. It frequently leaves conſiderable marks, which are much larger than thoſe of the ſmall-pox; as large, I have meaſured ſome, as a ſilver threepence.

Extract of a Letter from the Reverend Herman Drew to Dr. Pearſon; dated Wootton, September 7, 1798.

Having an opportunity, I communicated your queries relative to the cow-pox to Mr. Dolling, an inoculator at Blandford, who is in very extenſive practice, and I am favoured with the following anſwers:

I can only ſay, I have inoculated for the ſmall-pox many hundreds, that ſaid they had had the cow-pox; very few of them took the infection, ſo as to produce the ſmall-pox; and thoſe, I am inclined to think, deceived themſelves in regard to their having had the cow-pox. I inoculated ſeven children for one Perſen, the eldeſt not ten years old; five of thoſe children the mother had made play with cows teats that had the cow-pox, and they received infection from the cow; theſe five children had not ſmall-pox, the other two had.

Mr. Juſtins, a farmer at Yetminſter, in Dorſet, inoculated his wife and children with matter taken from the teats of a cow that had the cow-pox; in about a week from the time of inoculation, their arms were very much inflamed; the patients very ill; the man ſo much alarmed as to call in medical aſſiſtance (Mr. Meach of Cerne); the patients ſoon got well; they have ſince been inoculated for the ſmall-pox by Mr. Trobridge of Cerne; but did not have it.

Extract of a Letter from Dr. R. Pulteney, to Dr. Pearſon; dated Blandford, July 14, 1798.

I have never heard of any being affected with the diſeaſe, except ſuch have milked the cows or handled the udders.

The ſame perſon informed me, that of ſeven children he had inoculated for the ſmall-pox, five had been previouſly afflicted with with the cow-pox, and purpofely, by being made to handle the teats and udders of infected cows, in confequence of which they fuffered the diftemper. Thefe five, after inoculation for the fmall-pox, did not ficken; the other two had the diftemper.

It is well known in Hampfhire, Dorfet, Somerfet, and Devon. I alfo know that it is not uncommon in Leicefterfhire, and other midland counties; but dairy men keep it as fecret as poffible, as it is difreputable to the cleanlinefs of the produce.

Extract of a Letter from Mr. W. Dolling to Dr. Pearfon; dated Chittle, April 9, 1802.

The farmer alluded to in Dr. Pulteney's letter to you, who inoculated his wife and children with matter taken from the teat of a cow, and the perfon mentioned in Mr. Drew's letter, viz. Mr. Juftins, is the fame perfon; both Dr. Pulteney's and Mr. Drew's intelligence came from me. I am not at this time certain as to the year, but believe it was in or before the year 1786; the farmer is ftill living, of whom I can have the particulars.

Extract of a Letter from the Rev. Herman Drew to Dr. Pearfon; dated Abbots, near Honiton, Devon, April 11, 1802.

I cannot inform you at what period Mr. Juftins of *Yetminster* (not Axminfter) inoculated his family; but I have no doubt but it was previous to Dr. Jenner's practice. I have by this poft communicated to Sir William Elford a curious fact, which came to my knowledge yefterday; that, above twenty years ago, a woman inoculated her children with matter taken from the cow, on the point of a large needle.

Extract of a Letter from Mr. Nicholas Bragge, to Sir William Elford, Bart. (a Member of your Committee), dated Axminfter, April 12th, 1802.

It is now more than thirty years ago that I firft made experiments, and proved that the vaccine difeafe was a prefervative againft the fmall pox; and it is, I believe, more than twenty years ago, that, through the Rev. Herman Drew, I acquainted Sir George Baker with the obfervations and experiments I had then made, which I am certain Sir George will readily acknowledge. Unhappily, an accident by fire deprived me of having recourfe to them now, but my memory will fupply me with enough to convince you, that Dr. Jenner is not the perfon entitled to the reward that may be thought deferving for fuch difcovery. It is now, I believe, twenty years ago, that Mrs. Randall, the wife of a refpectable farmer in the parifh of of Whitechurch, near Lynne, in Dorfetfhire (who is at this time a tenant to Lady Caroline Damer, in the fame parifh for which I have been concerned as an apothecary for the poor ever fince I have been in bufinefs) inoculated herfelf, and three or four children for it; and thofe children, who have long arrived at manhood, have fince inoculated their friends and neighbours whenever an opportunity has offered.

Extract of a Letter from the Rev. Herman Drew, to Sir William Elford, Bart. dated Abbotts, near Honiton, April 1ft, 1802.

Dr. Edward Jenner has undoubtedly very great merit in bringing Vaccine Inoculation into practice, but he is no more the difcoverer of the cow-pox and its effects than I am. Nearly twenty years ago, I wrote fheets of paper to Sir George Baker on this diforder, and I know not what occafioned his laying afide his intention of publifhing his inveftigations; he had had a previous correfpondence with Dr. Pulteney of Blandford on the fubject. Sir George defired me to inoculate with matter taken from the cow, but my endeavours to find out where I might get matter, were for a long time unfuccefsful, owing to the fecrecy of the farmers, whofe dairies were infected with fuch a filthy ulcerous diftemper, it would have marred the fale of their butter, &c.

About fourteen years ago, I difcovered an infected cow, and, by defire of Sir George Baker, I applied to Mr. Bragge, a Surgeon of Axminfter, to attempt Inoculation; but unfortunately we were too late, as the diforder was fo far abated in the animal, that the matter had loft its activity. We made ufe, however, of a diffolution of the fcabs in warm water, but without fuccefs.

When Dr. Jenner publifhed his obfervations, he was followed by Dr. Pearfon of Leicefter-fquare, who was introduced by Sir George to a correfpondence with me on this fubject, and he repeatedly confeffes in print his obligations to me for information; no one can have an higher opinion of the good effects of the Vaccine Inoculation than I have, it has occupied my thoughts for years, and nothing but Horace's advice "ne futor ultra crepidam" has checked me from the ufe of the infected lancet or faturated cotton. Entre nous, I have had a little fuccefsful practice.

A letter alfo from William Tucker, Efquire, of Coryton in Devonfhire, to Sir William Elford, Bart. dated Coryton, April 12th, 1802, ftates, That Mr. Bragge, twenty years ago, proved the efficacy, and with great affiduity recommended the practice of Vaccine Inoculation; that Mr. N. Bragge, through the Rev. Herman Drew, furnifhed Sir George Baker with a variety variety of papers in proof of its being a ſure guard againſt variolous infection; and that Dr. Jenner's ſuperior merit conſiſted in having effected the introduction of Vaccine Inoculation, and in having alſo, as it is ſaid, aſcertained the means of diſcriminating the real from the ſpurious diſeaſe.

When Dr. Pearſon had finiſhed his evidence, Dr. Jenner inquired if he might be permitted to aſk Dr. P. one queſtion, which Admiral Berkeley would have aſked if he had been preſent. The Committee ſaid, "Certainly not; but you may propoſe your queſtion to the Chairman, and we will conſider how far it is proper to be aſked.

"It is," ſaid Dr. Jenner, "with what deſign did Dr. Pearſon pay a viſit to the confidential clerk of the Committee?"

Dr. Pearſon roſe to anſwer it, but the Committee inſiſted upon the room being cleared immediately.

In the anti-chamber Dr. P. told us he only wanted a copy of the petition.

In a few minutes the Committee ſent out their clerk to inform us that they would hear no more evidence, but proceed to the making up their Report. We of courſe went home to dinner "with what appetite we may."

As a farther commentary on the above evidence, and an additional proof of our impartiality, we give the following card from Dr. P. to Dr. B.

*Leiceſter Square, July* 4, 1802.

Dr. PEARSON preſents his compliments to Dr. Bradley, deſires to inform him that the Honourable Committee for Dr. Jenner's Petition, have not, as aſſerted in the Medical and Phyſical Journal, p. 26, laſt Number, publiſhed "*the whole of the contravening evidence*," but a ſmall part of that delivered by himſelf on the claim for diſcovery of inoculation is given; and no part of that reſpecting the diſcovery of particular facts, and the eſtabliſhment of the practice, is inſerted in the printed Report; neither is the evidence of others, on one point or other, wholly inſerted. If the Editors have reſerved Dr. Pearſon's evidence for the claſs of contravening evidence, of which they propoſe to give a ſtatement in their next Number, it ſeems improper they ſhould do ſo,* becauſe it contains the only, or the

---

* If the Editors have miſtaken the intention of Dr. P. in bringing forward his evidence; if Dr. P. meant to ſerve Dr. Jenner, inſtead of injuring him, they are ready to acknowledge that his evidence ſhould not have been placed among that which they conſider as *contravening*. But if the Editors have fallen into this miſtake, they believe it is a miſtake common to them, and all who heard it.

the fingle teftimony which fpecifically and diftinctly affords the ground for the declaration of the claim afferted by the Honour-able Committee on the point of Difcovery of Inoculation.

Dr. Pearfon defires further to notice, that he does not confider the extracts printed (for it is indeed an extract) by the Honourable Committee, gives for the moft part, the fenfe of his evidence; but here he difclaims any imputation on the Members of the Committee; nor is it a matter of regret to him, if the ftatement of the Report is fo reprefented as to ferve the intereft of the Petitioner, fince he differs only in opinion with refpect to the ground on which the claim ought to have been founded. Dr. Pearfon apprehends that the grounds propofed by him to the Honourable Committee on one of the three claims fet forth by the Petitioner, would have been as honourable and profitable to the Petitioner as thofe declared in the Report; with the advantage to boot of allowing due honour to other perfons.

Dr. Pearfon muft, in duty to the public, remark, that it does not feem fair to confider the Report of the Honourable Committee in the light of a ftrictly juft inveftigation and competent judgment of the rights of the Petitioner and of the merits of the Vaccine Inoculation:

1ft. Becaufe medical practitioners only are competent to fuch inquiries. Hence the late inquiry in a Committee of the Houfe of Commons was *coram non judice.*

2d. Becaufe, to ufe the words of a moft able and impartial Member of the Honourable Committee,* "He always received the Report of a Committee with fome degree of jealoufy, as he confidered them in the light of nominees on a committee to try the merits of a controverted election, as being friends of the petitioners." †

Dr. Pearfon takes the liberty of making one more remark, which is, that the Appendix containing the evidence being unu-ufually fhort, the public will perhaps be jealous on account of the Editors making a felection of teftimonies; efpecially as the Committee themfelves have already only made a felection from a body of evidence; and as the Editors have thought proper to deliver their opinion refpecting the decifion previous to laying the evidence before the public.

---

* Mr. Bankes.

† By thefe two obfervations I cannot be fuppofed to mean to arraign the juftice, or even to queftion the wifdom of delegating fuch powers to a Committee of the Houfe of Commons, which may be allowed very confiftently with the truth of the above remarks.

*Observations*

